# GATA-2 Transduces LPS-Induced *il-1β* Gene Expression in Macrophages via a Toll-Like Receptor 4/MD88/MAPK-Dependent Mechanism

**DOI:** 10.1371/journal.pone.0072404

**Published:** 2013-08-06

**Authors:** Tsu-Tuan Wu, Yu-Ting Tai, Yih-Giun Cherng, Tyng-Guey Chen, Chien-Ju Lin, Ta-Liang Chen, Huai-Chia Chang, Ruei-Ming Chen

**Affiliations:** 1 Graduate Institute of Medical Sciences and Center of Excellent Cancer Research, Taipei Medical University, Taipei, Taiwan; 2 Section of Respiratory and Critical Care Medicine, Department of Internal Medicine, Taipei County Hospital, Taipei, Taiwan; 3 Department of Anesthesiology, Wan-Fang Hospital, Taipei Medical University, Taipei, Taiwan; 4 Department of Anesthesiology, Shuang-Ho Hospital, Taipei Medical University, Taipei, Taiwan; 5 Anesthetics and Toxicology Research Center, Department of Anesthesiology, Taipei Medical University Hospital, Taipei, Taiwan; 6 Cell Physiology and Molecular Image Research Center, Wan-Fang Hospital, Taipei Medical University, Taipei, Taiwan; University Medical Center Freiburg, Germany

## Abstract

Lipopolysaccharide (LPS) is a critical factor for inducing acute lung injury. GATA-2, a transcription factor, contributes to the control of cell activity and function. Exposure of RAW 264.7 cells to LPS induced interleukin (IL)-1β mRNA and protein expression and GATA-2 translocation from the cytoplasm to nuclei in concentration- and time-dependent manners. A bioinformatic search revealed that GATA-2-specific binding elements exist in the 5’-promoter region of the *il-1β* gene. LPS could enhance the transactivation activity of GATA-2 in macrophages. Knocking-down translation of GATA-2 mRNA using RNA interference significantly alleviated LPS-induced IL-1β mRNA and protein expression. As to the mechanism, transfection of toll-like receptor (TLR) 4 small interfering (si)RNA into macrophages concurrently decreased LPS-caused increases in nuclear GATA-2 levels. Sequentially, treatment with myeloid differentiation factor 88 (MyD88) siRNA decreased LPS-induced phosphorylation of mitogen-activated protein kinases (MAPKs) kinase 1/2 and subsequent translocation of GATA-2. Reducing MAPK activities using specific inhibitors simultaneously decreased GATA-2 activation. Furthermore, exposure of primary macrophages to LPS significantly increased the transactivation activities of GATA-2 and IL-1β mRNA and protein expression. Transfection of GATA-2 siRNA inhibited LPS-induced IL-1β mRNA expression. Results of this study show that LPS induction of *il-1β* gene expression in macrophages is mediated by GATA-2 via activation of TLR4, MyD88, and MAPKs.

## Introduction

Gram-negative bacterium-induced acute lung injury and acute respiratory distress syndrome are common complications that occur in intensive care unit patients with acute pulmonary infections, frequently leading to mortality and morbidity [1,2]. Lipopolysaccharide (LPS), an outer membrane component of gram-negative bacteria, was implicated as one of the major causes of acute lung injury and septic shock [3,4]. In the lower respiratory tract responsive to LPS stimulation, alveolar macrophages are the first-line immune cells encountered by inhaled organisms [5]. As a result, alveolar macrophages play pivotal roles in a host’s cellular defense against infection and tissue injury in human lungs [6,7]. When activated by bacterial infection, alveolar macrophages can overproduce massive amounts of inflammatory cytokines, triggering progressive immune reactions [8,9]. Among them, interleukin (IL)-1β is reported to functionally induce acute edematous lung injury that resembles changes in the lungs of patients with lung injury due to acute respiratory distress syndrome [10]. Thus, understanding the mechanisms of LPS-induced *il-1β* gene expression will be beneficial to finding strategic treatments of acute lung injury.

Toll-like receptors (TLRs) are type-I transmembrane proteins with extracellular domains comprised largely of leucine-rich repeats and intracellular signaling domains [11]. In macrophages, TLR4 is a major receptor responsible for LPS stimulation [12,13]. When associated with LPS, the TLR4 complex can trigger cascade activation of intracellular adaptor myeloid differentiation factor 88 (MyD88) and mitogen-activated protein kinase (MAPK) kinases (MEK) 1/2 [14,15]. After that, phosphorylated MEKs sequentially stimulate phosphorylation of MAPK family proteins and certain transcriptional factors [16]. Activator protein (AP)-1 and nuclear factor (NF)-κB are two typical transcription factors that were reported to act by LPS stimulation to induce inflammatory cytokine genes [17,18]. Meanwhile, growing lines of evidence show that there are other transcription factors, such as rel, C/EBP, Ets, IRF3, and Egr, that are involved in activating LPS-inducible gene expressions [19,20]. Since LPS-induced pulmonary inflammation may be lethal to acute-lung-injury patients, investigating potential transcription factors, beside AP-1 and NF-κB, that participate in the LPS-involved inflammatory reaction is crucial for diagnosing and treating acute lung injury and acute respiratory distress syndrome.

GATA-DNA-binding proteins (GATAs) are a family of transcriptional regulators containing two zinc fingers with a Cys-X2-Cys-X17-Cys-X2-Cys motif that directly binds to the nucleotide sequence, element (A/T) GATA(A/G) [21]. In general, GATA-1, -2, and -3 are known to regulate critical events in hematopoietic lineages, while GATA-4, -5, and -6 are mainly expressed in non-hematopoietic tissues, including the heart and gut [22]. However, our previous study demonstrated that GATA-3 is expressed in primary osteoblasts and mediates cell survival signals [23]. In addition, GATA-3 can transcriptionally regulate *interleukin* gene expression in T-helper 2 cells, which controls cell differentiation and mediates allergic inflammation [24]. In LPS-induced septic shock, GATA-2 was shown to regulate tissue factor pathway inhibitor gene expression in human umbilical vein endothelial cells [25]. GATA-2 was also shown to be involved in macrophage differentiation [26]. However, the roles of GATAs in LPS-stimulated macrophage activation are still unknown. Our preliminary results revealed that GATA-2 was detected in peripheral and peritoneal macrophages. A previous study done in our lab demonstrated that LPS induced IL-1β messenger (m) RNA and protein expressions by macrophages [27]. Moreover, searching with a bioinformatics approach disclosed the existence of GATA-specific DNA motifs in the promoter region of the *il-1β* gene. Thus, in this study, we evaluated the roles of GATA-2 in LPS-induced *il-1β* gene expression and the possible mechanisms using murine macrophage-like RAW 264.7 cells and primary peritoneal macrophages as the experimental models.

## Materials and Methods

### Cell culture and drug treatment

A murine macrophage cell line, RAW 264.7, was purchased from the American Type Culture Collection (Rockville, MD, USA). RAW 264.7 cells were cultured in RPMI 1640 medium (Gibco-BRL, Grand Island, NY, USA) supplemented with 10% inactivated fetal calf serum (FCS), L-glutamine, penicillin (100 IU/ml), and streptomycin (100 µg/ml) in 75-cm^2^ flasks at 37 °C in a humidified atmosphere of 5% CO_2_. RAW 264.7 cells were allowed to grow to confluence prior to drug treatment.

LPS, purchased from Sigma (St. Louis, MO, USA), was extracted from *Escherichia coli* serotype O26: B6. LPS was dissolved in phosphate-buffered saline (PBS) (0.14 M NaCl, 2.6 mM KCl, 8 mM Na_2_HPO_4_, and 1.5 mM KH_2_PO_4_). RAW 264.7 cells were exposed to 1, 10, and 100 ng/ml for 1, 3, and 6 h. Inhibitors of MAPKs, including SB203580 for P38MAPK, SP600125 for c-Jun N-terminal kinase (JNK), and PD98059 for extracellular signal-regulated kinase (ERK), were purchased from Sigma (St. Louise, MO, USA). Prior to the addition of drugs, RAW 264.7 cells were washed with PBS, and non-adherent cells were removed. Control cells received PBS only.

### Isolation of peritoneal macrophages and immunocytochemical identification

Peritoneal macrophages from mice were prepared following a previously described method [28]. This investigation conformed to the *Guide for the Care and Use of Laboratory Animals* published by the US National Institutes of Health (NIH Publication no. 85-23, revised 1996), and all procedures were pre-approved by the Institutional Animal Care and Use Committee of Taipei Medical University, Taipei, Taiwan. ICR mice (20~25 g) were purchased from the Laboratory Animal Center, College of Medicine, National Taiwan University (Taipei, Taiwan). Casein was intraperitoneally injected into mice at a dose of 40 mg per kg body weight. After treatment for 96 h, resident macrophages were obtained from the peritoneal cavity by an injection with PBS. Following centrifuging and washing, macrophages were seeded in RPMI 1640 medium supplemented with 10% FCS, L-glutamine, penicillin (100 IU/ml), and streptomycin (100 µg/ml) in 75-cm^2^ flasks at 37 °C in a humidified atmosphere of 5% CO_2_. Cells were identified using an immunocytochemical analysis of F4/80, a macrophage-specific marker, as described previously [29].

### Reverse-transcription (RT) and quantitative polymerase chain reaction (PCR) assays

Messenger (m) RNA from macrophages exposed to drugs were prepared for RT-PCR and quantitative PCR analyses of interleukin (IL)-1β and β-actin. Oligonucleotides for the PCR analyses of IL-1β and β-actin were designed and synthesized by Clontech Laboratories (Palo Alto, CA, USA). The oligonucleotide sequences of the upstream and downstream primers for these mRNA analyses were respectively 5'-ATGGCAACTGTTCCTGAACTCAACT-3' and 5'-CAGGAC-AGGTATAGAATTCTTTCCTTT-3' for IL-1β [30] and 5'-GTGGGCCGCTCTAGGCACCAA-3' and 5'-CTCTTTGATGTCACGCACGATTTC-3' for β-actin [31]. The PCR was carried out using 35 cycles of 94 °C for 45 s, 60 °C for 45 s, and 72 °C for 2 min. The PCR products were loaded onto a 1.8% agarose gel containing 0.1 µg/ml ethidium bromide, and electrophoretically separated. DNA bands were visualized and photographed under ultraviolet-light exposure. The intensities of the DNA bands in the agarose gel were quantified with the aid of the UVIDOCMW vers. 99.03 digital imaging system (UVtec, Cambridge, UK). A quantitative PCR analysis was carried out using iQSYBR Green Supermix (Bio-Rad, Hercules, CA, USA) and the MyiQ Single-Color Real-Time PCR Detection System (Bio-Rad) as described previously [23].

### Enzyme-linked immunosorbent assay (ELISA)

The amounts of IL-1β in the culture medium of macrophages exposed to LPS were determined following the method described previously [13]. Briefly, after drug administration, the culture medium was collected and centrifuged. Levels of IL-1β in the culture medium were quantified following the standard protocols of the ELISA kits purchased from R&D Systems (Minneapolis, MN, USA).

### Prediction of transcription factor binding sites

The specific DNA binding sites and motifs of transcription factor GATA-2 in the promoter region of *il-1β* gene were searched and predicted using the TFSEARCH : Searching Transcription Factor Binding Sites (ver 1.3; http://www.rwcp.or.jp/papia/). The power of TFSEARCH program written by Yutaka Akiyama (Parallel Application TRC Laboratory, Real World Computing Partnership, Japan) is directly owed to the TRANSFAC databases developed at GBF-Braunschweig, Germany [32].

### Confocal microscopic analysis of GATA-2 translocation

GATA-2 in macrophages was recognized by a specific antibody and visualized using confocal microscopy following a previously described method [33]. Briefly, after drug treatment, macrophages were fixed with a fixing reagent (acetone: methanol, 1: 1) at -20 °C for 10 min. Following rehydration, cells were incubated with 0.2% Triton X-100 at room temperature for 15 min. The rabbit polyclonal antibody used in this study was generated against mouse GATA-2 (Santa Cruz Biotechnology, Santa Cruz, CA, USA). Immunodetection of GATA-2 in macrophages was carried out at 4 °C overnight. After washing, cells were sequentially reacted with the second antibodies and biotin-SP-conjugated AffiniPure goat anti-rabbit immunoglobulin G (IgG) (Jackson ImmunoResearch, West Grove, PA, USA) at room temperature for 1 h. After washing, the third antibody with Cy3-conjugated streptavidin (Jackson ImmunoResearch) was added to macrophages and reacted at room temperature for 30 min. Nuclei of fixed macrophages were stained with TOTO-3 (Molecular Probes, Eugene, OR, USA) at 37 °C for 30 min. A confocal laser scanning microscope (Model FV500, Olympus, Tokyo, Japan) was used for sample observation. Images were acquired and quantified using FLUOVIEW software (Olympus).

### Extraction of nuclear proteins and immunodetection

The amounts of nuclear transcription factors were quantified following a previously described method [33]. Briefly, after drug treatment, nuclear extracts of macrophages were prepared. Protein concentrations were quantified by a bicinchonic acid protein assay kit (Pierce, Rockford, IL, USA). Nuclear proteins (50 µg/well) were subjected to sodium dodecylsulfate polyacrylamide gel electrophoresis (SDS-PAGE), and transferred to nitrocellulose membranes. After blocking, nuclear GATA-2 was immunodetected using a rabbit polyclonal antibody against mouse GATA-2 (Santa Cruz Biotechnology). Cellular proliferating cell nuclear antigen (PCNA) was immunodetected using a mouse monoclonal antibody against mouse PCNA (Sigma St, Louis, MO, USA) as the internal control. Intensities of the immunoreactive bands were determined using a digital imaging system (UVtec).

### Electrophoretic mobility shift assay (EMSA)

An EMSA was performed using a Dig gel shift kit (Roche Diagnostics, Mannheim, Germany) as described previously [13]. Briefly, GATA-2 consensus oligonucleotides, purchased from Santa Cruz Biotechnology, were labeled with digoxigenin (DIG). The nuclear extract (10 µg) was allowed to react with DIG-labeled oligonucleotides at room temperature for 25 min. The complex was subjected to non-denatured PAGE, and transferred to positively charged nylon membranes. After cross-linking at 120 mJ and blocking, the membranes were immunoreacted with anti-DIG-GATA-2. Following washing and chemiluminescent detection, the membranes were exposed to x-ray film. Intensities of the immunoreactive bands were determined using a digital imaging system (UVtec).

### GATA-2, TLR4, and MyD88 knockdown

Translation of GATA-2, TLR4, and MyD88 mRNA in macrophages was knocked down using an RNA interference (RNAi) method as described previously [30]. GATA-2, TLR4, and MyD88 small interfering (si) RNAs were purchased from Santa Cruz Biotechnology, which is a pool of 3 target-specific 20~25-nt siRNAs designed to respectively knock down GATA-2, TLR4, and MyD88 expressions. GATA-2, TLR4, and MyD88 siRNAs were transfected into macrophages according to an siRNA transfection protocol provided by Santa Cruz Biotechnology. Briefly, after culturing macrophages in antibiotic-free RPMI medium at 37 °C in a humidified atmosphere of 5% CO_2_ for 24 h, the siRNA duplex solution, which was diluted in siRNA transfection medium (Santa Cruz Biotechnology), was added to the macrophages. After transfection for 24 h, the medium was replaced with normal RPMI medium, and macrophages were treated with drugs. Scrambled siRNA, purchased from Santa Cruz Biotechnology, was transfected to macrophages as a negative standard.

### Immunoblot analyses of TLR4, GATA-2, MyD88, and β-actin

Protein analyses were carried out as previously described [34]. After TLR4, GATA-2, and MyD88 siRNA treatment for 24 and 48 h, cell lysates were prepared in ice-cold radioimmunoprecipitation assay buffer (RIPA, 25 mM Tris-HCl (pH 7.2), 0.1% SDS, 1% Triton X-100, 1% sodium deoxycholate, 0.15 M NaCl, and 1 mM EDTA). Protein concentrations were quantified using a bicinchonic acid protein assay kit (Pierce, Rockford, IL, USA). Proteins (50 µg/well) were subjected to SDS-PAGE, and transferred to nitrocellulose membranes. After blocking, TLR4, GATA-2, and MyD88 were immunodetected using goat polyclonal antibodies against mouse TLR4, GATA-2, and MyD88 (Santa Cruz Biotechnology). Cellular β-actin was immunodetected using a mouse monoclonal antibody against mouse β-actin (Sigma) as the internal standard. These protein bands were quantified using a digital imaging system (UVtec) as described previously [34].

### Immunoblotting analyses of phosphorylated and non-phosphorylated MEK1/2

Cellular protein levels were immunodetected according a previously described method [35]. Briefly, after drug treatment, cell lysates were prepared in ice-cold RIPA buffer. Protein concentrations were quantified using a bicinchonic acid protein assay kit (Pierce). Proteins (50 µg per well) were subjected to SDS-PAGE, and transferred to nitrocellulose membranes. After blocking, phosphorylated MEK1/2 was immunodetected using a rabbit polyclonal antibody against phosphorylated residues of MEK1/2 (Cell Signaling, Danvers, MA, USA). Non-phosphorylated MEK1 was immunodetected as the internal control (Cell Signaling). These protein bands were quantified using a digital imaging system (UVtec).

### Immunoinhibition Assay

A goat polyclonal antibody against mouse TLR4 (Santa Cruz Biotechnology) were preincubated with macrophages for 30 min at 37 °C, then treated with LPS, and the nuclear extracts were prepared. The levels of nuclear GATA-2 were immunodetected. Nuclear PCNA was detected as the internal controls. These protein bands were quantified using a digital imaging system (UVtec).

### Statistical analysis

One-way ANOVA with the Bonferroni multiple-comparison test was used to compare IL-1β mRNA, GATA-2 translocation and transactivation, knock-down of GATA-2, TLR4, MyD88, and MEK1/2 phosphorylation in response to different treatments of LPS, siRNAs, or MAPK inhibitors. Values in the text are the mean ± SD. Differences were considered significant at *p* < 0.05.

## Results

### Toxicity of LPS to RAW 264.7 cells and peritoneal macrophages

Exposure of murine macrophage-like RAW 264.7 cells to 1, 10, and 100 ng/ml LPS for 1, 3, and 6 h did not affect cell viability (data not shown). After treatment with 1, 10, and 100 ng/ml LPS for 1, 3, and 6 h, the viability of primary peritoneal macrophages had not changed (data not shown).

### LPS induces IL-1β mRNA expression and activation of transcription factors

**Figure 1:**
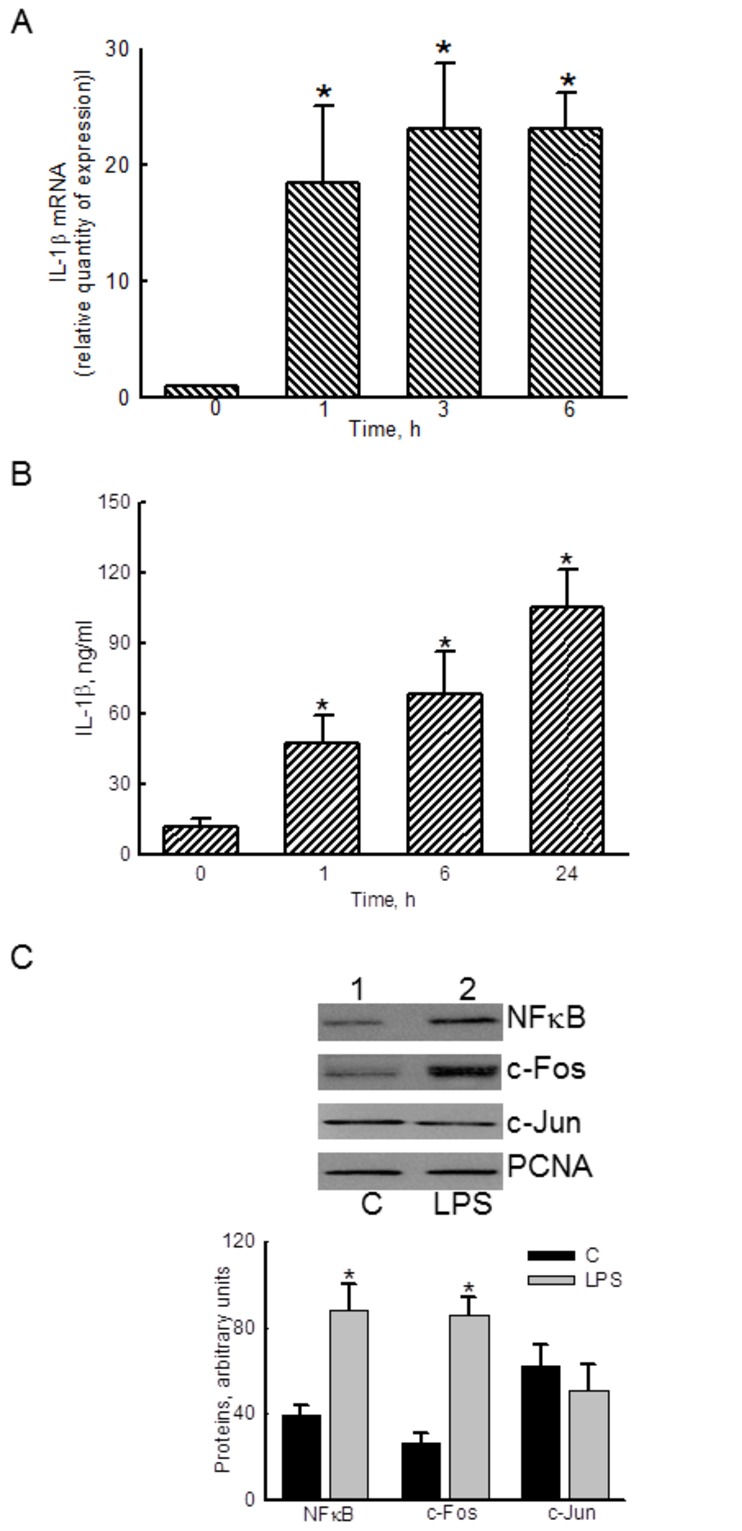
Effects of lipopolysaccharide (LPS) on the expression of interleukin (IL)-1β mRNA and protein as well as translocation of transcription factors. After exposure to 100 ng/ml LPS, the levels of IL-1β mRNA (A) and protein (B) in RAW 264.7 cells were determined using real-time PCR and ELISA analyses, respectively. Amounts of the nuclear transcription factors nuclear factor (NF)-κB and activator protein (AP)-1 were immunodetected (C, *top panels*). PCNA was measured as the internal control. These immunorelated proteins were quantified and statistically analyzed (*bottom panel*). The immunoblotting results shown are a representative of 6 experiments, and the other statistically analyzed results are a compilation of 6 replications. Each value represents the mean ± SD. An asterisk (*) indicates that the value significantly differed from the respective control, *p* < 0.05.

Exposure of RAW 264.7 cells to 100 ng/ml LPS for 1, 3, and 6 h caused significant 18-, 23-, and 23-fold inductions of IL-1β mRNA, respectively (Figure 1A). In comparison, the amounts of IL-1β protein in macrophages were significantly enhanced by 4-, 6-, and 9-fold following exposure to 100 ng/ml LPS for 1, 6, and 24 h (Figure 1B). Treatment of RAW 264.7 cells with LPS increased the levels of nuclear NFκB and c-Fos (Figure 1C, *top panels*, lane 2). However, amounts of c-Jun in RAW 264.7 cells were not changed by LPS. Nuclear PCNA was immunodetected as the internal standard. These protein bands were quantified and statistically analyzed (Figure 1C, *bottom panel*). LPS respectively increased levels of nuclear NF-κB and c-Fos by 2.3- and 3.3-fold.

### LPS stimulates translocation and transactivation of GATA-2

**Figure 2:**
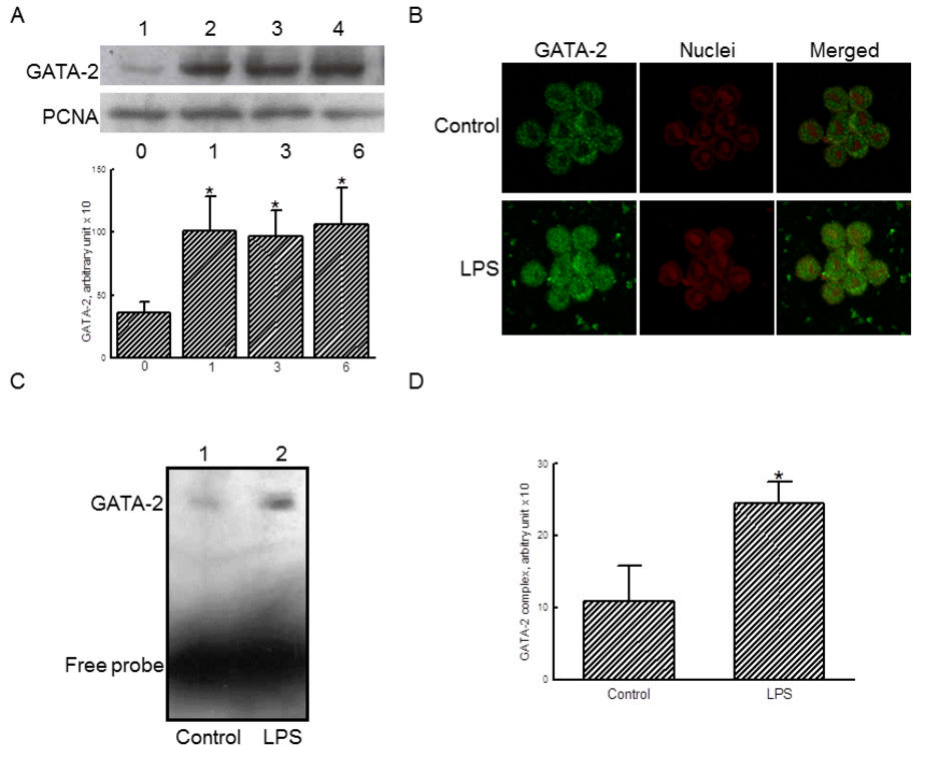
Effects of lipopolysaccharide (LPS) on the translocation and transactivation of GATA-2. RAW 264.7 cells were exposed to 100 ng/ml LPS for 1, 3, and 6 h. Amounts of nuclear GATA-2 were immunodetected (A, *top panel*). Levels of nuclear PCNA were measured as the internal control. These immunorelated proteins were quantified and statistically analyzed (*bottom panel*). RAW 264.7 cells were treated with 100 ng/ml LPS, the translocation of GATA-2 from the cytoplasm to nuclei were analyzed using confocal microscopy (B). The transactivation activity of GATA-2 was assayed using an EMSA analysis (C). These DNA-protein bands were quantified and statistically analyzed (D). The immunoblotting, confocal, and DNA-protein binding results shown are a representative of at least 3 experiments, and the other statistically analyzed results are a compilation of 6 replications. Each value represents the mean ± SD. An asterisk (*) indicates that the value significantly differed from the respective control, *p* < 0.05.

Results by a bioinformatic search reveal that there are 5 predicted binding sites of transcription factor GATA-2 located at -199, -547, -590, -916, and -1582 of the 5’-promoter region of the *il-1β* gene (Table 1). Treatment of RAW 264.7 cells with 100 ng/ml LPS for 1, 3, and 6 h enhanced the levels of nuclear GATA-2 in macrophages (Figure 2A, lanes 2-4). Nuclear PCNA was immunodetected as the internal standard. These protein bands were quantified and statistically analyzed (Figure 2A, *bottom panel*). After exposure to LPS for 1, 3, and 6 h, LPS caused significant 2.8-, 2.7-, and 3-fold augmentation in the translocation of GATA-2 from the cytoplasm to nuclei, respectively.

**Table 1:** The predicted binding sites and motifs of transcription factor GATA-2 in the 5’-promoter region of the *il-1β* gene.

Predicted DNA binding sites	
Locations	Motifs
-199 ^~^ -204	WGATAR
-547 ^~^ -552	YTATCW
-590 ^~^ -595	WGATAR
-916 ^~^ -921	WGATAR
-1582 ^~^ -1587	YTATCW

These DNA binding sites and motifs were searched and predicted using the TFSEARCH : Searching Transcription Factor Binding Sites (ver 1.3; http://www.rwcp.or.jp/papia/).

Analyses of confocal microscopy showed that exposure of RAW 264.7 cells to 100 ng/ml LPS for 1 h increased the levels of cytosolic GATA-2 (Figure 2B, *left panels*). After exposure to LPS, the amounts of GATA-2 in nuclei were apparently augmented (Figure 2B, *right panels*). In addition, the EMSA analysis further revealed that LPS enhanced the binding activity of nuclear extracts to GATA-2 consensus oligonucleotides (Figure 2C). Free probes were quantified as the internal standard. These protein-DNA bands were quantified and statistically analyzed (Figure 2D). LPS caused a significant 2.2-fold increase in the transactivation activity of GATA-2.

### GATA-2 participates in LPS-induced IL-1β mRNA expression

**Figure 3:**
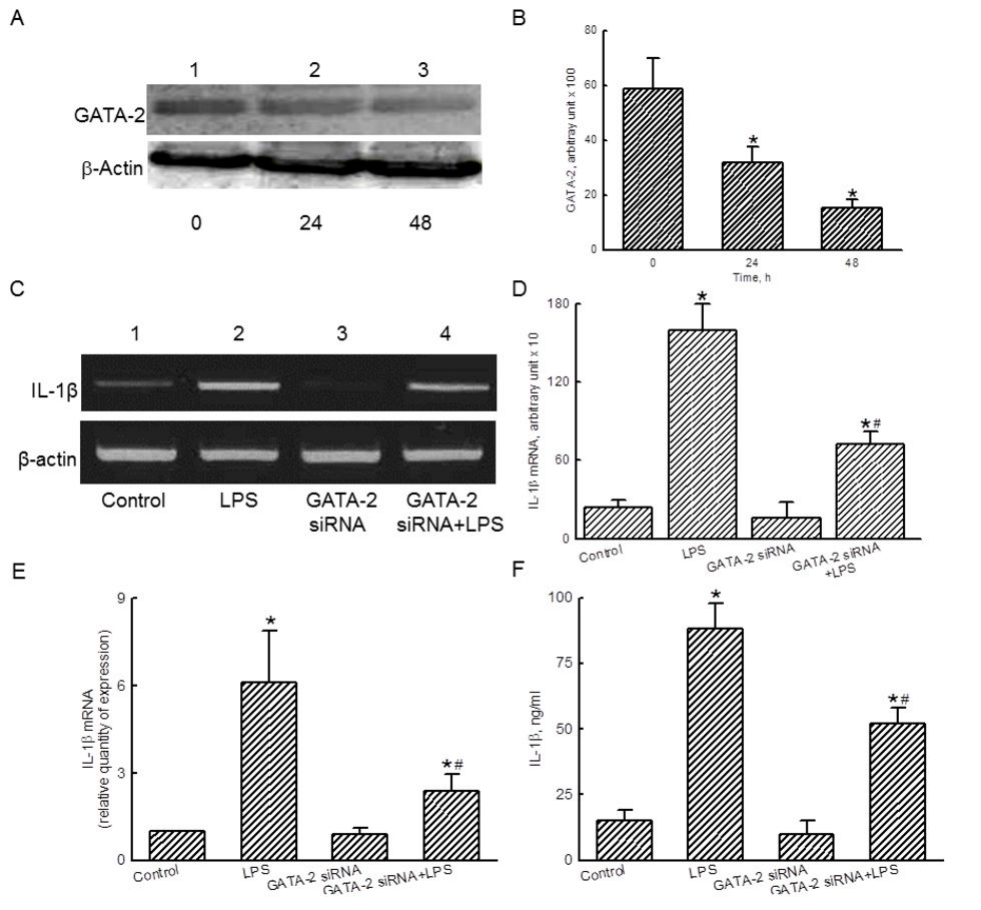
Roles of GATA-2 in lipopolysaccharide (LPS)-induced interleukin (IL)-1β mRNA expression. RAW 264.7 cells were subjected to GATA-2 small interference (si) RNA for 24 and 48 h. Levels of GATA-2 were immunodetected (A, *top panel*). β-Actin was measured as the internal control (*bottom panel*). These protein bands were quantified and statistically analyzed (B). RAW 264.7 cells were exposed to LPS, GATA-2 siRNA, and a combination of GATA-2 siRNA and LPS. Amounts of IL-1β mRNA were determined using an RT-PCR analysis (C, *top panel*). β-Actin mRNA was analyzed as the internal control (*bottom panel*). These DNA bands were quantified and statistically analyzed (D). A real-time PCR analysis was conducted to confirm the roles of GATA-2 (E). Effects of GATA-2 siRNA on LPS-induced IL-1β production were determined by ELISA (F). The immunoblotting results shown are a representative of 6 experiments, and the other statistically analyzed results are a compilation of 6 replications. Each value represents the mean ± SD. An asterisk (*) and pound sign (^#^) indicate that a value significantly (*p* < 0.05) differed from the respective control and LPS-treated group, respectively.

To determine the role of GATA-2 in LPS-induced IL-β mRNA expression, GATA-2 siRNA was transfected into RAW 264.7 cells. After treatment with GATA-2 siRNA for 24 and 48 h, translation of GATA-2 in RAW 264.7 cells was obviously downregulated (Figure 3A, *top panel*, lanes 2 and 3). β-Actin was immunodetected as the internal control (Figure 3A, *bottom panel*). These protein bands were quantified and statistically analyzed (Figure 3B). Transfection of GATA-2 siRNA to RAW 264.7 cells for 24 and 48 h significantly decreased levels of GATA-2 by 46% and 75%, respectively.

In untreated RAW 264.7 cells, low levels of IL-1β mRNA were detected (Figure 3C, *top panel*, lane 1). After exposure to LPS for 6 h, IL-1β mRNA was induced (lane 2). Transfection of GATA-2 siRNA did not affect IL-1β mRNA expression (lane 3), but actually attenuated LPS-induced IL-1β mRNA synthesis (lane 4). Amounts of β-actin mRNA were analyzed as the internal standard (Figure 3C, *bottom panel*). These DNA bands were quantified and analyzed (Figure 3D). LPS induced IL-1β mRNA expression by 6.6-fold. However, GATA-2 siRNA significantly lowered LPS-induced IL-1β mRNA expression by 55% (Figure 3D). Analyses of real-time PCR further showed that knocking down GATA-2 expression caused a 60% inhibition of LPS-induced IL-1β mRNA expression (Figure 3E). After treatment with LPS, the levels of IL-1β were increased by 5.8-fold (Figure 3F). Transfection of GATA-2 siRNA did not change IL-1β levels but caused a significant 43% decrease in LPS-induced IL-1β production.

### TLR4 is involved in regulating GATA-2 translocation

**Figure 4:**
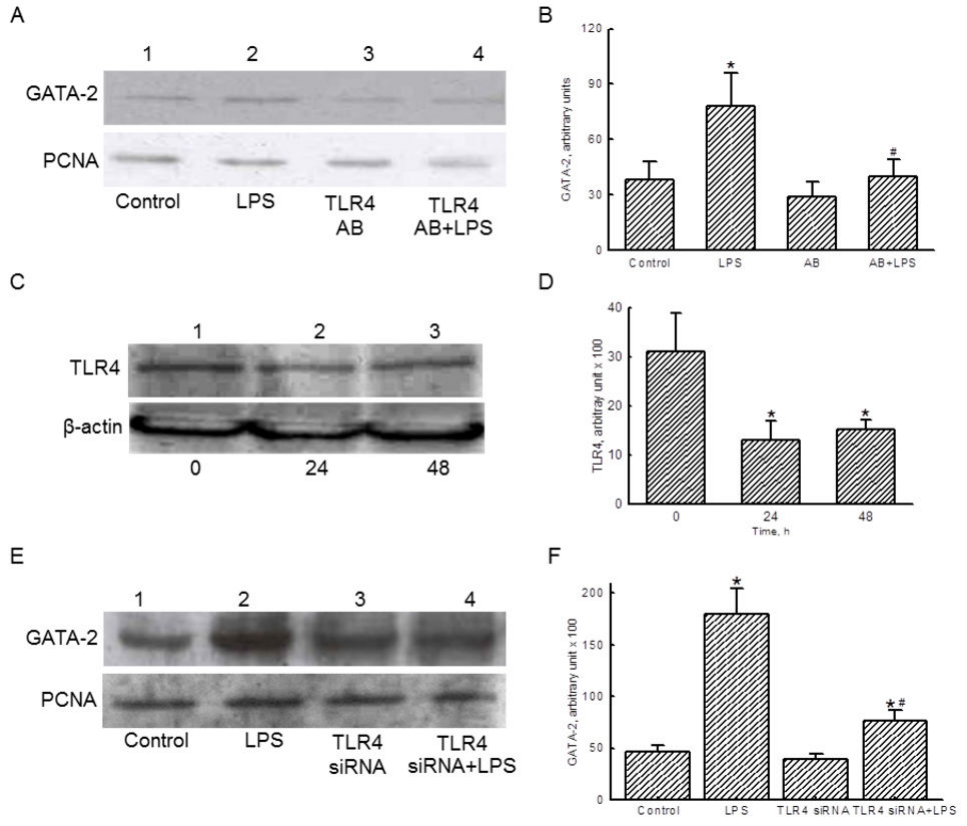
Roles of Toll-like receptor (TLR)-4 in lipopolysaccharide (LPS)-induced translocation of GATA-2. RAW 264.7 were pretreated with TLR4 antibody (AB) for 30 min, and then treated with LPS. Levels of nuclear GATA-2 were immunodetected (A, *top panel*). PCNA was determined as the internal controls (*bottom panel*). These immunorelated protein bands were quantified and statistically analyzed (B). RAW 264.7 cells were transfected to TLR-4 small interference (si) RNA for 24 and 48 h. Levels of TLR-4 were immunodetected (C, *top panel*). β-Actin was measured as the internal control (*bottom panel*). These protein bands were quantified and statistically analyzed (D). RAW 264.7 cells were exposed to LPS, TLR4 siRNA, and a combination of TLR4 siRNA and LPS. Amounts of GATA-2 were immunodetected (E, *top panel*). PCNA was measured as the internal control (*bottom panel*). These protein bands were quantified and statistically analyzed (F). The immunoblotting results shown are a representative of 6 experiments, and the other statistically analyzed results are a compilation of 6 replications. Each value represents the mean ± SD. An asterisk (*) and pound sign (^#^) respectively indicate that the value significantly (*p* < .05) differed from the respective control and LPS-treated group.

Exposure of Raw 264.7 cells to LPS increased the levels of nuclear GATA-2 (Figure 4A, *top panel*, lane 2). TLR4 antibody did not affect nuclear GATA-2 levels but decreased LPS-induced translocation of GATA-2 (lanes 3 and 4). PCNA was immunodetected as the internal controls (Figure 4A, *bottom panel*). These protein bands were quantified and analyzed (Figure 4B). Pretreatment with TLR4 antibody significantly decreased LPS-enhanced nuclear GATA-2 levels by 51%.

Transfection of TLR4 siRNA to RAW 264.7 cells for 24 and 48 h reduced translation of this membrane receptor (Figure 4C, *top panel*, lanes 3 and 4). β-Actin was immunodetected as the internal control (Figure 4C, *bottom panel*). Treatment with TLR4 siRNA for 24 and 48 h respectively caused significant 58% and 52% decreases in the amounts of this membrane receptor in RAW 264.7 cells (Figure 4D). Exposure of RAW 264.7 cells to LPS increased levels of nuclear GATA-2 (Figure 4E, *top panel*, lane 2). TLR4 siRNA did not influence nuclear GATA-2 amounts (lane 3), but alleviated LPS-induced translocation of this transcription factor from the cytoplasm to nuclei (lane 4). Nuclear PCNA was immunodetected as the internal control (Figure 4E, *bottom panel*). These immunorelated protein bands were quantified and statistically analyzed (Figure 4F). LPS increased the level of nuclear GATA-2 by 3.9-fold. Transfection of TLR4 siRNA caused a significant 58% reduction in LPS-induced GATA-2 translocation.

### MyD88 contributes to LPS-induced GATA-2 activation

**Figure 5:**
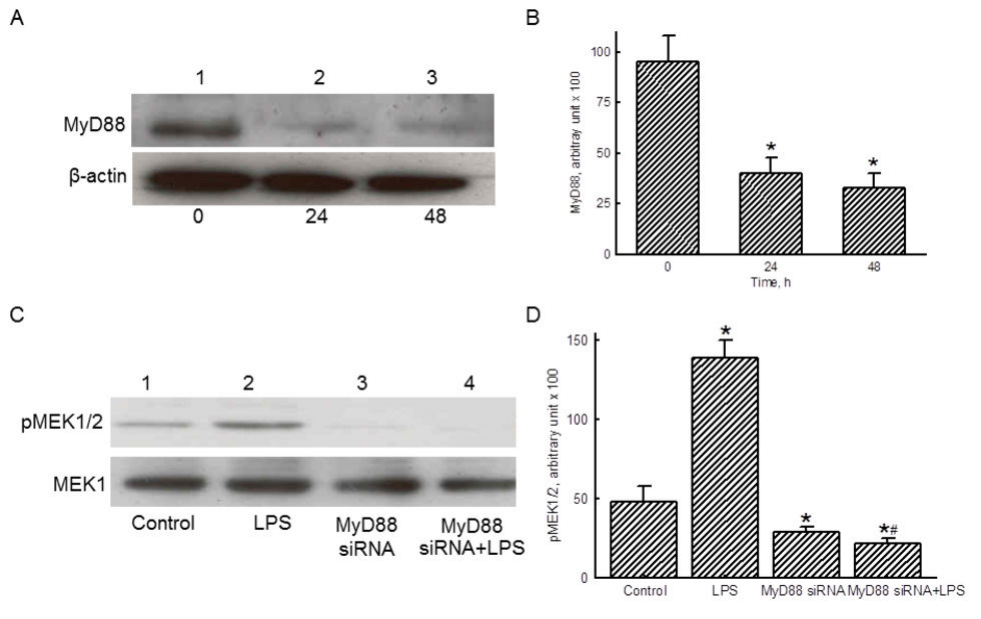
Roles of MyD88 in lipopolysaccharide (LPS)-induced activation of MEK1/2. RAW 264.7 cells were subjected to MyD88 small interfering (si) RNA for 24 and 48 h. Levels of MyD88 were immunodetected (A, *top panel*). β-Actin was measured as the internal control (*bottom panel*). These protein bands were quantified and statistically analyzed (B). RAW 264.7 cells were exposed to LPS, MyD88 siRNA, and a combination of MyD88 siRNA and LPS. Amounts of phosphorated MEK1/2 were immunodetected (C, *top panel*). MEK1 was measured as the internal control (*bottom panel*). These protein bands were quantified and statistically analyzed (D). The immunoblotting results shown are a representative of 6 experiments, and the other statistically analyzed results are a compilation of 6 replications. Each value represents the mean ± SD. An asterisk (*) and pound sign (#) respectively indicate that the value significantly (*p* < 0.05) differed from the respective control and LPS-treated group.

Treatment of RAW 264.7 cells with MyD88 siRNA for 24 and 48 h downregulated the amounts of this adaptor (Figure 5A, *top panel*, lanes 2 and 3). β-Actin was immunodetected as the internal control (Figure 5A, *bottom panel*). These protein bands were quantified and analyzed (Figure 5B). Transfection of MyD88 siRNA to RAW 264.7 cells for 24 and 48 h significantly decreased the amounts of this adaptor by 58% and 67%, respectively.

LPS could induce phosphorylation of MEK1/2 in RAW 264.7 cells (Figure 5C, *top panel*, lane 2). Transfection of MyD88 siRNA significantly inhibited levels of phosphorylated MEK1/2 (lane 3), and decreased LPS-induced phosphorylation of MEK1/2 (lane 4). MEK1 was immunodetected as the internal control (Figure 5C, *bottom panel*). These protein bands were quantified and analyzed (Figure 5D). Exposure to LPS caused a significant 2.4-fold increase in amounts of phosphorylated MEK1/2, but MyD88 siRNA completely attenuated such enhancement.

### MAPKs mediate LPS-stimulated GATA-2 translocation

**Figure 6:**
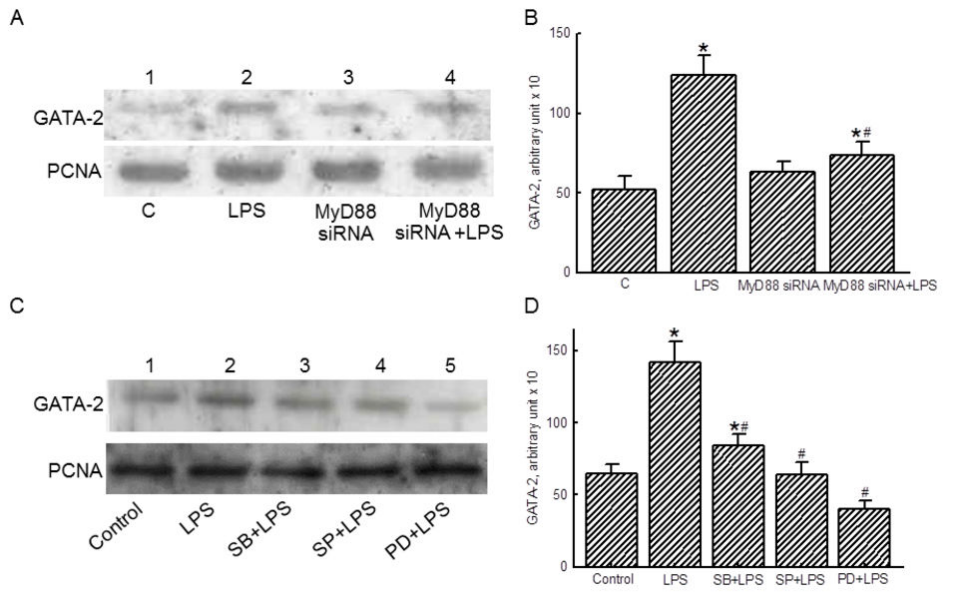
Effects of MyD88 small interference (si) RNA and MAPK inhibitors on translocation of GATA-2. RAW 264.7 cells were exposed to lipopolysaccharide (LPS), MyD88 siRNA, and a combination of MyD88 siRNA and LPS. Amounts of GATA-2 were immunodetected (A, *top panel*). PCNA was measured as the internal control (*bottom panel*). These protein bands were quantified and statistically analyzed (B). RAW 264.7 cells were pretreated with 10 µM MAPK inhibitors, including SB203580 (SB), SP600125 (SP), and PD98059 (PD), for 1 h and then exposed to LPS. Nuclear GATA-2 was immunodetected (C, *top panel*). Amounts of PCNA were measured as the internal control (*bottom panel*). These protein bands were quantified and statistically analyzed (D). The immunoblotting results shown are a representative of 6 experiments, and the other statistically analyzed results are a compilation of 6 replications. Each value represents the mean ± SD. An asterisk (*) and pound sign (^#^) respectively indicate that the value significantly (*p* < 0.05) differed from the respective control and LPS-treated group.

Exposure of RAW 264.7 cells to LPS increased amounts of nuclear GATA-2 (Figure 6A, *top panel*, lane 2). Transfection of MyD88 siRNA did not change the basal levels of nuclear GATA-2 but decreased LPS-induced translocation of GATA-2 from the cytoplasm to nuclei (lanes 3 and 4). Nuclear PCNA was immunodetected as the internal control (Figure 6A, *bottom panel*). These protein bands were quantified and analyzed (Figure 6B). LPS increased translocation of GATA-2 by 2.8-fold, but treatment with MyD88 siRNA completely inhibited this augmentation.

Nuclear lysates were prepared for following analyses. Pretreatment with the MAPK inhibitors, SB203580, SP600125, and PD98059, obviously decreased LPS-induced GATA-2 translocation to nuclei (Figure 6C, *top panel*, lanes 3-5). Nuclear PCNA was immunodetected as the internal control (Figure 6C, *bottom panel*). These protein bands were quantified and analyzed (Figure 6D). Pretreatment with these MAPK inhibitors completely inhibited LPS-induced translocation of GATA-2 from the cytoplasm to nuclei.

### Role of GATA-2 in regulating LPS-induced IL-1β mRNA expression is further confirmed in primary macrophages

**Figure 7:**
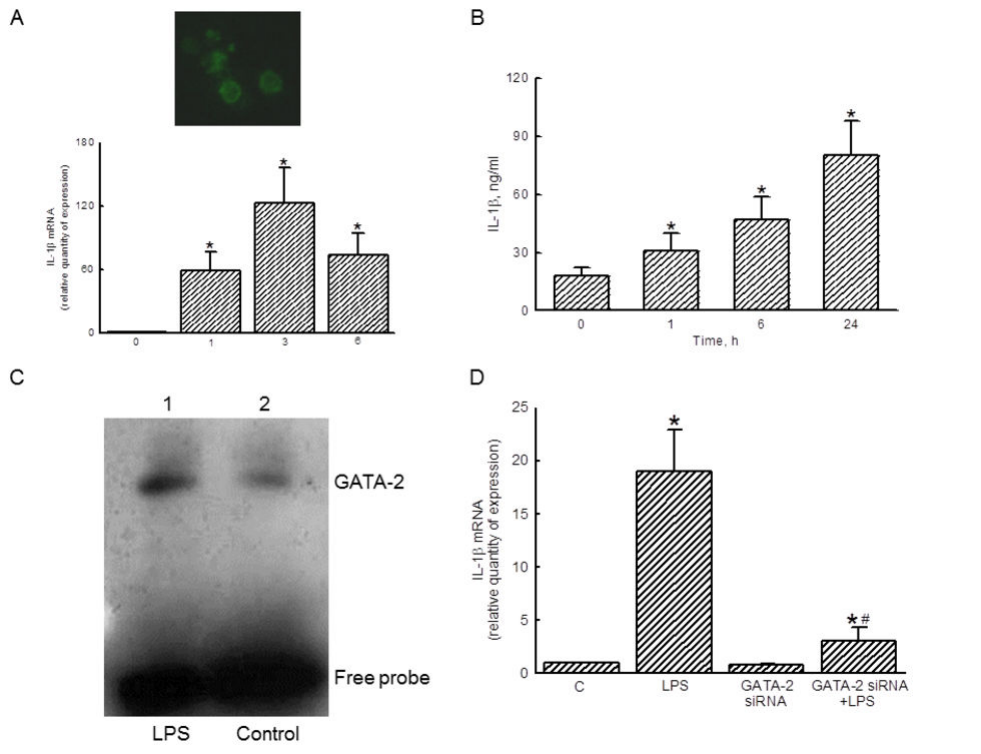
Roles of GATA-2 in lipopolysaccharide (LPS)-induced interleukin (IL)-1β mRNA expression in primary macrophages. Peritoneal macrophages prepared from mice were identified using an immunocytochemical confocal analysis of the F4/80 protein, a macrophage-specific marker (A, *top panel*). After exposure to 100 ng/ml LPS for 1, 3, and 6 h, the levels of IL-1β mRNA in primary macrophages were quantified using a real-time PCR analysis (A, *bottom panel*). The amounts of IL-1β protein were determined using ELISA (B). The transactivation activity of GATA-2 was assayed using an EMSA analysis (C). Primary macrophages were treated with LPS, GATA-2 siRNA, and a combination of GATA-2 siRNA and LPS, and IL-1β mRNA were quantified (D). The immunoblotting, confocal, and DNA-protein binding results shown are a representative of 6 experiments, and the other statistically analyzed results are a compilation of 6 replications. Each value represents the mean ± SD. An asterisk (*) and pound sign (^#^) respectively indicate that the value significantly (*p* < 0.05) differed from the respective control and LPS-treated group.

To confirm the roles of GATA-2 in primary macrophages, peritoneal macrophages were isolated from ICR mice (Figure 7). An immunocytochemical analysis of F4/80 revealed that more than 90% of the isolated cells were macrophages (Figure 7A, *top panel*). In untreated primary macrophages, the expression of IL-1β mRNA was very slight (Figure 7A, *bottom panel*). Exposure to LPS for 1, 3, and 6 h caused great induction of IL-1β mRNA expression. As well, the results from ELISA analyses revealed that exposure to 100 ng/ml LPS for 1, 6, and 24 h, the levels of IL-1β protein were augmented by 72%, 261%, and 444%, respectively (Figure 7B). The EMSA analysis revealed that LPS increased the binding activity of nuclear extracts to GATA-2 consensus oligonucleotides (Figure 7C). Transfection of GATA-2 siRNA significantly inhibited LPS-induced IL-1β mRNA expression in peritoneal macrophages by 84% (Figure 7D).

## Discussion

This study shows that LPS simultaneously induced translocation of c-Fos and NFκB and expression of IL-1β mRNA and protein. c-Fos is a member of the AP-1 family proteins [13]. Our previous studies demonstrated that following LPS stimulation, translocation of AP-1 and NFκB from the cytoplasm to nuclei and their transactivation activities were respectively enhanced, and expressions of the *il-1β*, *tumor necrosis factor-α*, and *inducible nitric oxide synthase* genes were simultaneously induced [13,30,31,35]. Interestingly, this study showed that the amounts of GATA-2 concurrently increased in peripheral RAW 264.7 cell and peritoneal macrophages. Our bioinformatic search revealed the existence of GATA-2-specific DNA binding elements in the promoter region of the *il-1β* gene. This study shows that LPS can improve the transactivation activity of GATA-2. In comparison, knocking down the translation of GATA-2 caused noteworthy attenuation of LPS-induced GATA-2 translocation and subsequent IL-1β mRNA and protein expression. Thus, in addition to AP-1 and NFκB, this study showed that GATA-2 is involved in regulating LPS-induced IL-1β mRNA and protein expressions. In general, GATA-2 is known to regulate critical events in hematopoietic lineages [22]. However, a previous study validated that in patients undergoing elective cardiopulmonary bypass surgery, polymorphisms in the proximal promoter region of the *il-10* gene are associated with *in vivo* and *ex vivo* LPS sensitivity via a GATA-dependent mechanism [36]. The present study provides further *in vitro* evidence to corroborate the role of GATA-2 in Gram-negative bacterium-triggered immune defense.

MyD88 is a key adapted molecule that transduces TLR4-initiated intracellular signals [37]. The present results indicate that MyD88 knocking down attenuated LPS-induced GATA-2 translocation. TLR4 is shown to induce GATA-2 activation and IL-1β mRNA protein syntheses. Hence, MyD88 activation in LPS-treated macrophages may be due to an upstream change in TLR4’s conformation. Analyses of protein kinases further revealed the roles of MyD88 in regulation of MEK1/2 phosphorylation in LPS-treated macrophages. MEK1/2 can act as a downstream target of the TLR4/MyD88 complex [38]. As a result, the association of TLR4 with MyD88 can phosphorylate MEK1/2, and then induce GATA-2 translocation. Once its role is accomplished, MyD88 is recycled for use by other TLRs. A therapeutic strategy for defeating sepsis by misleading MyD88 was proposed [39]. MyD88 can activate the transcription factors, AP-1 and NF-κB [17,18]. However, this study further reports a de novo role of MyD88 in galvanizing GATA-2 activation and subsequent *il-1β* gene expression.

LPS can enhance phosphorylation of MEK1/2. In comparison, knocking down MyD88 decreased LPS-induced MEK1/2 phosphorylation and GATA-2 translocation. Thus, MEK1/2 can mediate TLR4/MyD88-triggered GATA-2 activation. In addition, MEKs are upstream enzymes that can phosphorylate downstream MAPKs, including ERK1/2, JNK1/2, and p38MAPK [16]. Supplementary immunoblotting analyses done in the present study disclosed the roles of these MAPKs in GATA-2 activation. A previous study demonstrated that upon IL-3 stimulation of hematopoietic cells, GATA-1 was strongly phosphorylated at residue serine 26 by a MAPK-dependent pathway [40]. GATA-1 and GATA-2 have similar structures and functions [21,22]. During erythropoiesis, a transcription factor GATA-1/GATA-2 balance is normally present [22,41]. Hence, MAPKs may directly phosphorylate GATA-2 and then stimulate its translocation from the cytoplasm to nuclei. Our present results indicate the effects of LPS on improvement of GATA-2 transactivation activity. Therefore, LPS can cause cascade activation of TLR4, MyD88, MEK1/2, MAPKs, and GATA-2, and consequently induces IL-1β mRNA expression.

Primary macrophages were identified by an immunocytochemical analysis of F480, a macrophage-specific marker [29]. LPS is also shown to induce IL-1β mRNA and protein expression in primary macrophages. Bysani et al. reported that the plasma concentration of LPS in a patient with fatal *Klebsiella pneumoniae* sepsis was 25 ng/mL [42]. Meanwhile, the concentration of LPS used in this study was 100 ng/ml, and under such a condition, this endotoxin did not influence macrophage morphology or viability. Thus, LPS at 100 ng/ml induced IL-1β mRNA expression but did not cause insults to peritoneal macrophages. As well, LPS can increase the transactivation activity of GATA-2 in primary macrophages. Transfection of GATA-2 siRNA did not cause cytotoxicity to peritoneal macrophages but significantly lessened LPS-induced IL-1β mRNA expression. Thus, like the action of GATA-2 on macrophage-like RAW 264.7 cells, we further showed that GATA-2 can transduce LPS-triggered inflammatory signals to induce *il-1β* gene expression in primary macrophages.

In summary, this study shows that LPS could induce IL-1β mRNA and protein expression in RAW 264.7 cells. As shown by analyses of EMSA and confocal microscopy, exposure of RAW 264.7 cells to LPS increased translocation and transactivation activities of GATA-2. In comparison, reducing GATA-2 synthesis attenuated LPS-induced IL-1β mRNA expression. As to the mechanism, certain molecules, including TLR4, MyD88, MEK1/2, and MAPKs, were involved in LPS-induced GATA-2 activation and *il-1β* gene expression. Furthermore, the role of GATA-2 in LPS-induced IL-1β mRNA and protein expression was confirmed in primary macrophages. Therefore, according to the present results, we suggest that GATA-2 can mediate LPS-induced inflammatory signals via cascade activation of TLR4, MyD88, and MEK1/2. In our laboratory, we are investigating the molecular mechanisms about how GATA-2 regulates *il-1β* gene expression, using certain methodologies including chromatin immunoprecipitation assay, cloning of the 5’-promoter region, and the site-directed mutagenesis method to determine the exact binding position of GATA-2. In addition, the roles of GATA-2 in regulating activation of alveolar macrophages in animals with acute lung injury are also validated in our laboratory.
